# Research Centers Collaborative Network Workshop on Sex and Gender Differences in Aging

**DOI:** 10.1093/geroni/igac055

**Published:** 2022-08-21

**Authors:** Tina E Brinkley, Shana D Stites, Holly C Hunsberger, Carrie A Karvonen-Gutierrez, Mengting Li, C Elizabeth Shaaban, Roland J Thorpe, Stephen B Kritchevsky

**Affiliations:** Department of Internal Medicine, Section on Gerontology and Geriatric Medicine, Wake Forest School of Medicine, Winston-Salem, North Carolina, USA; Department of Psychiatry, University of Pennsylvania, Philadelphia, Pennsylvania, USA; Department of Foundational Science and Humanities, Rosalind Franklin University of Medicine and Science, North Chicago, Illinois, USA; Department of Epidemiology, University of Michigan School of Public Health, Ann Arbor, Michigan, USA; School of Nursing, Institute for Health, Health Care Policy and Aging Research, Rutgers, The State University of New Jersey, New Brunswick, New Jersey, USA; Department of Epidemiology, Graduate School of Public Health, University of Pittsburgh, Pittsburgh, Pennsylvania, USA; Department of Health, Behavior, and Society, Johns Hopkins Bloomberg School of Public Health, Baltimore, Maryland, USA; Department of Internal Medicine, Section on Gerontology and Geriatric Medicine, Wake Forest School of Medicine, Winston-Salem, North Carolina, USA

**Keywords:** Biology, Epidemiology, Life course, Public health

## Abstract

Aging affects men and women differently; however, the impact of sex and gender on the aging process is not well understood. Moreover, these 2 concepts are often conflated, which further contributes to a lack of clarity on this important issue. In an effort to better understand the relevance of sex and gender in aging research, the Research Centers Collaborative Network sponsored a 1.5-day conference on sex and gender differences in aging that brought together key thought leaders from the 6 National Institute on Aging center programs. The meeting included sessions on comparing males and females, pathophysiological differences, sex/gender in clinical care, and gender and health in the social context. Presenters from a wide array of disciplines identified opportunities for multidisciplinary research to address current gaps in the field and highlighted the need for a more systematic approach to understanding the how and why of sex/gender differences, as well as the health implications of these differences and the sex/gender biases that affect clinical treatment and outcomes. This article summarizes the proceedings of the workshop and provides several recommendations to move the field forward, such as better data collection tools to assess the intersection of sex and gender in epidemiological research; a life course perspective with attention to fetal/developmental origins and key life stages; innovative animal models to distinguish contributions from sex hormones versus sex chromosomes; and integration of sex/gender into teaching and clinical practice. Ultimately, successful implementation of these recommendations will require thoughtful investigations across the translational spectrum and increased collaborations among those with expertise in sex and gender differences.

**Translational Significance:** Biological sex and gender influence health and disease during the aging process. However, our understanding of the underlying causes and consequences of sex and gender differences, which are likely rooted in fetal development, emerge during key life stages, and compound over the life course, remains incomplete. This article summarizes proceedings from a workshop on sex and gender differences in aging and provides recommendations for future research and clinical care.

In June 2019, the Research Centers Collaborative Network (RCCN) sponsored a 1.5-day workshop in Marina Del Rey, CA that focused on sex and gender differences in aging. This workshop brought together key thought leaders from each of the 6 National Institute on Aging center programs, including the Alzheimer’s Disease Research Centers (ADRCs), Centers on the Demography and Economics of Aging (CDEA), Claude D. Pepper Older Americans Independence Centers (OAICs), Nathan Shock Centers of Excellence in the Basic Biology of Aging, Resource Centers for Minority Aging Research (RCMARs), and Roybal Centers for Translational Research on Aging. The primary goal of the workshop was to facilitate a series of discussions on the biological, pathological, psychosocial, and clinical factors that contribute to sex and gender differences with advancing age. The present article highlights key themes of the workshop and describes opportunities for multidisciplinary research to address current gaps in the field, as well as recommendations to move the field forward. The workshop agenda, participants, and slides can be viewed online at: https://www.rccn-aging.org/sex-and-gender-rccn-workshop. Presenters and attendees are listed in the Online Supplementary Material.

## Defining Sex and Gender

Sex and gender are terms that are often used interchangeably; however, they are notably different as first recognized by psychologist Rhoda Unger in 1979 (1). Neither sex nor gender is binary despite often being treated dichotomously in research. Sex can be defined as a biological status of being male, female, or intersex. Gender, by contrast, consists of a range of enacted roles and behaviors that occur in historical and sociocultural contexts. While the American Psychological Association (2), the World Health Organization (3), and the Federal Drug Administration (4) all define gender in slightly different terms, it is clear that gender is a social construct influenced by societal and cultural expectations and norms and is shaped by the individual environment and lived experiences. Given the growing attention to health disparities across men and women, it is also clear that sex interacts with gender to influence health and disease, and that this interaction must be addressed in order to achieve health equity with aging (5).

## Studying Sex Differences in Aging

Sex differences have been widely reported, in large part due to several landmark cases (ie, the 1993 National Institutes of Health [NIH] Revitalization Act and the 21st Century Cures Act), Institute of Medicine reports, and NIH policy changes over the last 25 years (6,7). Collectively, these activities acknowledge the importance of sex, stipulating that women must be included in all clinical research, unless there is a clear and compelling reason to exclude them, and that sex must be considered in the planning, conducting, analyzing, and reporting of all clinical trials. The incorporation of such guidelines across the translational spectrum can be used to inform regulatory policies and clinical care, ultimately paving the way for optimal health for everyone at every stage of life.

The two main questions asked when studying sex differences are why they exist and how they exist. From a biological standpoint, sex differences are generally caused by sex hormones and sex chromosomes. While the traditional theory of sex determination focuses on gonadal differentiation as the primary event that precedes all other sex differences, it is now evident that numerous X and Y chromosomal factors, including sex-determining genes and their downstream products (eg, gonadal hormones), interact with each other to determine biological sex (8). Given the numerous nonreproductive targets of gonadal hormones and the age-discordance between men and women for the loss of gonadal hormones, it is particularly important that aging research includes a focus on sex as a contributing factor. Studying sex differences across the spectrum of translational research and across the life span will help fill knowledge gaps regarding the biological contributions of sex to human health. Growing evidence points to the role of X-chromosome inactivation, which due to incomplete inactivation of ~23% of X-linked genes, leads to sex-biased gene expression and thereby sex differences in health and disease (9). In addition, the identification of Y-chromosome gene activity has caused a paradigm shift as we now know that the Y chromosome is not just responsible for sex determination and spermatogenesis. On the contrary, data from association studies have revealed that genetic variation within the Y chromosome may be a critical determinant of health and disease susceptibility in men (10).

Perhaps one of the most common comparisons made between males and females is in life expectancy. The existence of a female longevity bias is well-established, and this has been shown not just in humans, but across multiple mammalian species (11). However, there are notable exceptions, including the Brandt’s bat (12), which has an extreme male longevity bias, and laboratory mice, which have variable longevity both between and within species (11). This variability presents an opportunity to better understand biological sex differences in aging, and animal models make an important contribution in this regard. One innovative model that has been used to study sex differences is the Four Core Genotypes mouse, which allows for separation of health effects related to sex chromosomes versus gonadal hormones (13,14). Studies using this and other animal models such as the ovariectomized mouse with estradiol added back (OVX+E2), the estrogen receptor alpha knockout mouse, and the genetically heterogeneous (UM-HET3) mouse clearly show the role of sex-based genetics and hormones in determining not only longevity, but also differences in metabolic outcomes (15–18).

In humans, sex differences in mortality may be best understood by examining the morbidity process, which is characterized by early molecular/cellular changes that lead to the onset of risk factors indicating physiological dysregulation, followed by the onset of disease and loss of function. Finally, a state of frailty ensues whereby a loss of organ reserve and severe physiological deterioration ultimately lead to death (19). While most studies examine the development of morbidity in adulthood, growing evidence suggests that common aging-related health conditions and diseases such as cardiovascular disease, depression, and Alzheimer’s disease may have developmental origins. According to the prenatal stress-immune model, prenatal exposures can alter the fetal stress response circuitry in the brain in a sex-dependent manner via organizational effects of gonadal hormones, and this fetal programming helps set the stage for sex differences in the adult offspring (20–22). Indeed, data on human sex differences in age-related changes in bone and heart health, immune function, and cognition/brain health highlight the importance of studying predictors of health in males and females across the life span as certain differences are present throughout life, whereas others are only apparent during or after key life stages (eg, early developmental period, puberty, and menopause) (23–26).

## Studying Gender Differences in Aging

Studying gender differences is complex, and relatively little work has been done to investigate the health impact of gender differences in aging. This may be partly due to the conflation of sex and gender, as well as a lack of awareness of tools or access to resources to adequately assess the three main dimensions of gender: gender roles (ie, behavioral norms), gender identity (ie, a person’s sense of being male, female, nonbinary, etc.), and gender relations (ie, interpersonal relationships). These aspects of gender appear at an early age and are reinforced throughout life by cultural expectations about how men/boys and women/girls should think, feel, behave, and relate. Furthermore, gender is not static over the life course. In turn, gendered practices and expectations can spill over to bias the provision of healthcare and conduct of research and can have a significant public health impact.

In research, gender bias can affect how we assess clinical predictors and outcome variables, thereby introducing information bias that can lead to errors in results and incorrect interpretations of the data. For example, a study by Sheehan et al. demonstrated that the measurement properties of the instrumental activities of daily living (IADL) questionnaire do not account for differences in gendered expectations in household activities (eg, preparing food and handling finances), and this likely biases gender differences in health-related IADL limitations, which are reportedly higher in women (27). Another example is depression, which by most accounts is lower in men; however, multiple lines of evidence suggest that this may be due, in part, to gender biases in the reporting, diagnosis, and manifestation of depression (28). Furthermore, the lack of data collection on sexual orientation and gender identity means that the higher rates of disability, mental distress, and social isolation in sexual and gender minoritized (SGM) older adults, due to discrimination and minority stress, are likely underestimated and inadequately addressed (29).

In the context of clinical care, social and cultural norms about sex and gender shape not only who gets treated, but how care is delivered. Moreover, reports that the sex/gender of the physician, the sex/gender of the patient, and the combinations thereof, can influence outcomes like hospital readmissions, heart attacks, and even mortality, further highlight patient affects of gender bias in medicine (30,31). Gender also affects clinical endpoints for caregivers. Despite narrowing gender gaps in parental and spousal caregiving, female caregivers continue to be disproportionately affected by poor mental and physical health outcomes and financial instability, compared to male caregivers (32–35). While effects on caregiver health are moderated by individual differences in resources and vulnerabilities, such as socioeconomic status, prior health status, and level of social support, these factors are themselves affected by sex/gender disparities (36). In SGM communities, gender bias creates obstacles to accessing and receiving quality care, both in terms of medical treatment and support services, and this can have significant consequences on an individual’s health (29).

It should be noted that many important issues related to gender and aging are beyond the scope of this article. However, there are several recent reviews that provide a more extensive discussion of the historical, sociocultural, and political lens through which gender diversity has traditionally been viewed, as well as insight on how to better understand the needs of SGM older adults with intersecting identities and enhance services, clinical care, research and policy to address the numerous inequalities affecting this growing population (37–39).

## Summary, Future Directions, and Research Opportunities

Although much progress has been made to advance understanding of the biological contributions to sex-based differences with advancing age, the study of gender-based differences in aging is still nascent, as perceptions surrounding sexual orientation and gender identity have evolved tremendously over the last few decades. With this in mind, several themes emerged from the workshop, and opportunities to address a number of knowledge gaps were highlighted. As summarized in the following sections and in Table 1, greater attention to the intersection of sex/gender with age and other sociodemographic factors, studies that utilize life course approaches, relevant animal models, tools to assess sexual and gender diversity, and addressing health disparities will be critical in transforming the current landscape of research in sex and gender differences in aging.

**Table 1.** Recommendations for Future Research on Sex and Gender Differences in Aging

Identified Gaps	Specific Research Examples that Address the Unmet Need
Intersectionality	Considering the joint effects of sex, gender, race, ethnicity, geography, etc.
	Collecting survey and interview data from intergenerational and SGM families
	Investigating the role of institutions and public health policies in creating systems of discrimination
Life course	Assessing changes in biomarkers over time in healthy and pathological aging
	Studying sex-based and gender-based mechanisms linking longevity to resilience and vulnerability
	Incorporating hallmarks of aging and multiomics data in population-based studies
	Using a broad comparative approach with animal models that mimic human aging
	Connecting prenatal exposures and fetal programming to health outcomes in adult offspring
Tools/Resources	Establishing best practices for studying sex and gender differences in aging
	Identifying/developing validated, gender-based assessments for use in clinical research
	Evaluating medical education programs that integrate sex/gender differences into clinical decision making

*Note*: SGM = sexual and gender minoritized.

### Intersectionality

Intersectionality is a framework for understanding how social and political identities combine to create different modes of discrimination and privilege (40). Intersectional perspectives should focus on not only sex and gender, but also other key demographics like race/ethnicity, socioeconomic status, geography, and ancestry, which all can influence aging processes. It will also be important to understand the role that institutions, policies, and other structural forces play in intersectionality and how this ultimately affects aging in diverse populations. Additionally, more data from surveys and semistructured interviews collected within and across generations and families, especially traditionally understudied (eg, SGM) families, are needed to better assess how patterns of sex/gender biases contribute to cumulative (dis)advantage processes and how these processes reflect and influence health and health care disparities.

### Life Course Studies

Life course studies can be used to help address the need for rigorous investigations of sex/gender-specific delineation of relevant biomarkers and how they contribute to changes in health over time with both aging and disease. Such studies can also advance scientific understanding of sex-based mechanisms that potentially link longevity to resilience and vulnerability. Incorporating more biologically informed data, such as hallmarks of aging (eg, inflammation, epigenetics, telomere length, cell senescence, and mitochondrial function) and omics measures (eg, whole-genome sequencing, metabolic profiles, epigenomics, protein and RNA expression patterns) into prospective population-based studies may help to identify early changes that are predictive of sex/gender differences in health span and life span. Additionally, by combining longitudinal sampling of properly stored cells with advanced methodological techniques (eg, immune cell profiling); integrating omics, molecular, behavioral, imaging, environmental, and clinical data; and utilizing analytical techniques that appropriately test for sex/gender differences (eg, testing interactions and stratification vs adjustment) and the effects of aging (ie, modeling age as a nonlinear variable), we may be better able to capture the morbidity process for populations and for individuals and further elucidate factors contributing to sex/gender differences with aging.

### Animal Studies

Animal studies have an important role in understanding sex differences in the biology of longevity. A broad comparative approach is recommended as the basic biological principles underlying observed sex differences can only be understood by studying animals across a range of species. Animal models that mimic human aging, including reproductive hormone changes and pathophysiological processes, will be particularly useful for investigating sex-based mechanisms underlying a variety of age-related health outcomes. In addition, while more studies like the New England Family Study are needed to determine how prenatal exposures are transmitted during fetal development and subsequent life stages to produce sex differences, animal models of fetal programming are more feasible and may provide insight into opportunities for modulating the emergence of sex differences in humans. However, it should be noted that animal models are only useful for studying sex differences, underscoring the need for researchers to identify robust methodological techniques for studying gender and its interaction with sex.

### Gender-Based Assessments

The field would greatly benefit from better guidance and tools for studying gender differences in aging. A 2005 paper by Becker et al. presented a thorough summary and discussion of best practices for studying sex differences in animal and human research, including a logic tree to guide the systematic approach to asking relevant questions aimed at understanding the biological origins of sex differences in a given trait (41). A similar paper on gender would be useful to give investigators interested in pursuing research on gender differences as many resources as possible to enable them to better incorporate validated, gender-based assessments in their studies, along with a basic understanding of how to analyze and interpret the data. Such assessments could be particularly useful in characterizing social relationships within the household, workplace, and larger community, which may shed light on the psychosocial, cultural, and political contexts through which gender inequalities persist in older populations. Based on a recent report by Stites et al., it is clear that few aging-related cohort studies adequately measure and define sex and gender constructs (42). This limitation along with inconsistent data collection across studies impedes the inclusion and representation of sociocultural diversity in research samples. They also limit what researchers can understand about the influences of sex and gender on aging-related pathways and outcomes.

### Health Disparities

To reduce health disparities and improve health equity for older adults from SGM communities, it is imperative that we effectively translate new research findings to the bedside. It has previously been shown that better integration of sex and gender into clinical decision-making can be achieved simply by asking patient sex and gender, acknowledging sex-based variability in disease presentation, and recognizing potential limitations in diagnostic tests and questionnaires (43). In addition, a study by Huded et al. demonstrated that a systems-based approach to treating a common cardiovascular condition can reduce gender disparity in health care outcomes (44). These and other findings suggest that addressing sex and gender in health and health care will require new approaches at many intervention points, from the training of medical personnel to sex/gender-based medical treatments and public health strategies and regulations (5). More examples and evidence for the integration of sex and gender into clinical care will be needed to provide models of care that can be applied systematically across health care systems.

## Conclusions

There are numerous sex and gender-based differences that affect how men and women age. The complexity of these issues will require a concerted effort to incorporate sex and gender in research across the translational spectrum and make changes in teaching, clinical practice, and regulatory policies. Only by advancing understanding of the *how* and *why* of sex and gender differences in aging will researchers and clinicians be better able to reduce sex/gender disparities in health and health care in older adults.

## Supplementary Material

igac055_suppl_Supplementary_Material
