# Supplementary material for: Research Centers Collaborative Network Workshop on Sex and Gender Differences in Aging
Source: Innov Aging. 2022 Aug 21;6(7):igac055. doi: 10.1093/geroni/igac055 (PMC9579719; doi:10.1093/geroni/igac055)
Supplement: igac055_suppl_Supplementary_Material [file igac055_suppl_supplementary_material.docx]

**Online Supplementary Material: List of Workshop Organizers, Presenters, and Attendees**

**Presenters/Moderators**

Arthur P. Arnold, PhD

University of California, Los Angeles

Steven N. Austad, PhD

University of Alabama at Birmingham

Eileen M. Crimmins, PhD

University of Southern California

Margaret F. Doyle, PhD

University of Vermont

Dena B. Dubal, MD, PhD

University of California, San Francisco

Jill M. Goldstein, PhD

Harvard Medical School

Gail A. Greendale, MD

University of California, Los Angeles

Chyren Hunter, PhD

Office of Research on Women’s Health, NIH

Wendy Kohrt, PhD

University of Colorado, Denver

Neal Krause, PhD

University of Michigan, Ann Arbor

Stephen B. Kritchevsky, PhD

Wake Forest School of Medicine

Andrea Z. LaCroix, PhD

University of California, San Diego

Jeffrey S. Mogil, PhD

McGill University

Alison A. Moore, MD, MPH

University of California, San Diego

Kate Nagy

Office of Planning, Analysis, and Evaluation, NIA

James F. Nelson, PhD

University of Texas Health Center San Antonio

Cara Tannenbaum, MD, MSc

Université de Montréal

Roland J. Thorpe, PhD, MS

Johns Hopkins University

Debra J. Umberson, PhD

University of Texas at Austin

Elena Volpi, MD, PhD

University of Texas Medical Branch

**Early Career Travel Awardees**

Samira Abdulai-Saiku, PhD

University of California, San Francisco

Stacy L. Andersen, PhD

Boston University

Lynnette A. Averill, PhD

Yale University

Sarah Banks, PhD

University of California, San Diego

Helena M. Blumen, PhD, MS

Albert Einstein College of Medicine

DeAnnah Byrd, PhD

Wayne State University

Catherine Cheng, PhD

University of Texas Health Center San Antonio

Holly Hunsberger, PhD

Columbia University (Rosalind Franklin University of Medicine and Science)

Carrie Karvonen-Gutierrez, PhD, MPH

University of Michigan, Ann Arbor

Sungyhe Kim, MD

Wake Forest School of Medicine

Mengting Li, PhD

Rutgers, The State University of New Jersey

C. Elizabeth Shaaban, PhD, MPH

University of Pittsburgh

Shana D. Stites, PsyD, MS, MA

University of Pennsylvania

Ashley N. Turner, PhD

University of Alabama at Birmingham

**Other Attendees**

Hattie Herman

American Federation for Aging Research

Dyane McMachon

American Federation for Aging Research

Elizabeth Pritchett-Montavon

American Federation for Aging Research

Odette van der Willik

American Federation for Aging Research

Gary Chan, PhD

National Alzheimer's Coordinating Center, University of Washington

Merilee A. Teylan, MPH

National Alzheimer's Coordinating Center, University of Washington

Marino Bruce, PhD, MRSC, MDiv

Vanderbilt University

Allon Canaan, PhD

Yale University

Emily Davis

University of California, San Francisco

Deena S. Goldwater, MD, PhD

University of California, Los Angeles

Geidy E. Serrano, PhD

Banner Sun Health Research Institute

Albert Shieh, MD

University of California, Los Angeles
